# A method for accurate and reproducible specimen alignment for insertion tests of cochlear implant electrode arrays

**DOI:** 10.1007/s11548-023-02930-1

**Published:** 2023-05-19

**Authors:** Jakob Cramer, Georg Böttcher-Rebmann, Thomas Lenarz, Thomas S. Rau

**Affiliations:** 1 Department of Otolaryngology, Hannover Medical School, Carl-Neuberg-Str. 1, 30625 Hannover, Germany; 2 Cluster of Excellence EXC 2177/1 “Hearing4all”, Hannover Medical School, Hannover, Germany

**Keywords:** Specimen alignment, Electrode insertion tests, 3D printing, Porcine cochlea, Pose setting adapter

## Abstract

**Purpose:**

The trajectory along which the cochlear implant electrode array is inserted influences the insertion forces and the probability for intracochlear trauma. Controlling the trajectory is especially relevant for reproducible conditions in electrode insertion tests. Using ex vivo cochlear specimens, manual alignment of the invisibly embedded cochlea is imprecise and hardly reproducible. The aim of this study was to develop a method for creating a 3D printable pose setting adapter to align a specimen along a desired trajectory toward an insertion axis.

**Methods:**

Planning points of the desired trajectory into the cochlea were set using CBCT images. A new custom-made algorithm processed these points for automated calculation of a pose setting adapter. Its shape ensures coaxial positioning of the planned trajectory to both the force sensor measuring direction and the insertion axis. The performance of the approach was evaluated by dissecting and aligning 15 porcine cochlear specimens of which four were subsequently used for automated electrode insertions.

**Results:**

The pose setting adapter could easily be integrated into an insertion force test setup. Its calculation and 3D printing was possible in all 15 cases. Compared to planning data, a mean positioning accuracy of 0.21 ± 0.10 mm at the level of the round window and a mean angular accuracy of 0.43° ± 0.21° were measured. After alignment, four specimens were used for electrode insertions, demonstrating the practical applicability of our method.

**Conclusion:**

In this work, we present a new method, which enables automated calculation and creation of a ready-to-print pose setting adapter for alignment of cochlear specimens in insertion test setups. The approach is characterized by a high level of accuracy and reproducibility in controlling the insertion trajectory. Therefore, it enables a higher degree of standardization in force measurement when performing ex vivo insertion tests and thereby improves reliability in electrode testing.

**Supplementary Information:**

The online version contains supplementary material available at 10.1007/s11548-023-02930-1.

## Introduction

During cochlear implant (CI) surgery, the electrode array (EA) is inserted into the cochlea, where it electrically stimulates the auditory nerve using multiple small platinum electrode contacts. As the indication for a therapy with a CI has been expanded from deaf patients to patients with some extent of residual hearing [1, 2], the prevention of insertion trauma and the preservation of the sensitive structural elements of the cochlea gained relevance [3]. An important factor during EA insertion is the direction of entering the cochlear lumen, i.e., the insertion trajectory. In most cases, this trajectory is placed parallel to the centerline of the basal turn of the cochlea and within the central region of the scala tympani to avoid early contact between EA and intracochlear structures. Some studies suggest that angular deviations from this parallel trajectory can have a strong influence on the resulting insertion force [4] and can further lead to intracochlear trauma [5]. Moreover, multiple studies showed that the probability for intracochlear trauma is correlated to increased insertion forces [6–8]. Consequently, one focus in EA development is the reduction of forces occurring during the insertion process, which are directly linked to the design of the EA.

For a mechanical EA characterization or an investigation of causes for intracochlear trauma under controlled conditions, insertion tests with ex vivo specimens [6, 7, 9, 10] or artificial cochlear models (ACM) [11–14] are a standard procedure. In a typical test setup, an EA is automatically advanced into a cochlear specimen or an ACM, while the forces in insertion direction are measured. A common factor in most test setups is the coaxial alignment of the linear feed and the main force measurement axis of the sensor. Moreover, the desired trajectory into the cochlear specimen or ACM needs to be aligned to the insertion and force measurement axis, respectively. This is essential, since the investigation of relations between forces, trajectory and insertion trauma is only possible when the desired trajectory can be set reliably, as this ensures controlled conditions and thus reproducible and comparable results.

Using ACMs, which are mostly made from clear material, the desired insertion trajectory into the model is well known in advance, and therefore, the model can be aligned to the insertion axis simply by design of the test bench. The alignment and thus the standardization of insertion tests become more challenging when using ex vivo cochlear specimens. Here, the cochlear lumen has natural variations in shape [15, 16] and is embedded invisibly inside the bone. A common procedure for aligning ex vivo specimens is manual positioning using anatomical landmarks such as the round window and the bony structure surrounding the cochlea [7]. However, Torres et al. showed that manual alignment of the insertion axis toward a planned trajectory into the cochlea varies depending on the experience level of the surgeon and produces high angular deviations between these axes, even from CI experts (6°–7°) [17]. Furthermore, Yasin et al. showed that there is a wide variability among surgeons when choosing an insertion trajectory for EA insertion (21° ± 14.5°) [18]. These factors would lead to a non-negligible influence on the insertion test results, impeding controlled testing conditions. Especially when the specimen is cut down to the small area of the cochlea or otic capsule, the alignment difficulty increases, as important anatomical landmarks such as the facial recess get lost. Nevertheless, manual alignment is often used in this case [7, 9, 19], accepting the above-mentioned inaccuracies. Other works used advanced approaches from computer-assisted surgery. Huegl et al. combined manual alignment with additional guidance from an optical navigation system to provide a better control of the insertion trajectory [20]. However, this method needs a cost-intensive stereo-optic camera and additional reference markers, which need to be fixed to the small specimen and which introduce a higher weight to the sensitive force sensor. In a work of Kobler et al., the cochlear specimen was aligned by means of trajectory planning based on CT images, optical tracking of the specimen using a stereo-optic camera and positioning using a passive hexapod kinematic system [10]. While the use of a hexapod can further increase accuracy, this approach is highly complex and costly.

**Fig. 1:**
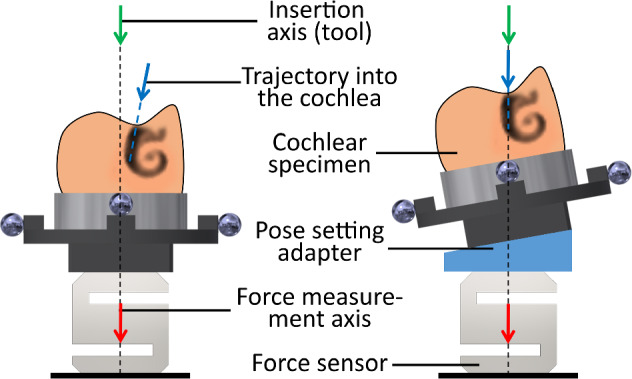
Basic idea of the pose setting method; left: mismatching trajectory into the cochlea and insertion axis; right: alignment of the axes using a pose setting adapter

Recently, we proposed a simple method that enables specimen alignment in insertion test setups in a precise and reproducible way [21]. This stereotactic approach is based on individual, 3D-printed pose setting adapters (PSA), which are mounted between the force sensor and the specimen to ensure accurate alignment (Fig. 1). However, a remaining drawback of this method is the creation of the PSA, which is based on a parametric computer-aided design (CAD) model controlled by several Excel spreadsheets which was susceptible to user errors. In addition, the method was never tested with real cochlear specimens in a structured and substantial study.

Therefore, this study covers the following aims: First, we wanted to improve the alignment method with a new algorithm that automatically creates a 3D printable file of the PSA directly from the planned trajectory points in order to increase the robustness of the process. This simplification was supposed to enable faster and easier PSA creation independent from external CAD software to make the method less error-prone. The second objective was to evaluate the alignment accuracy of this advanced method in a realistic application. For this reason, porcine cochlear specimens (*n* = 15) were used, as they are easily available and are said to be similar to the human cochlear anatomy [22], albeit a bit smaller. Lastly, insertion tests into four of the aligned porcine cochlear specimens were conducted as a proof of concept for the complete testing method.

## Material and methods

### General workflow

**Fig. 2:**
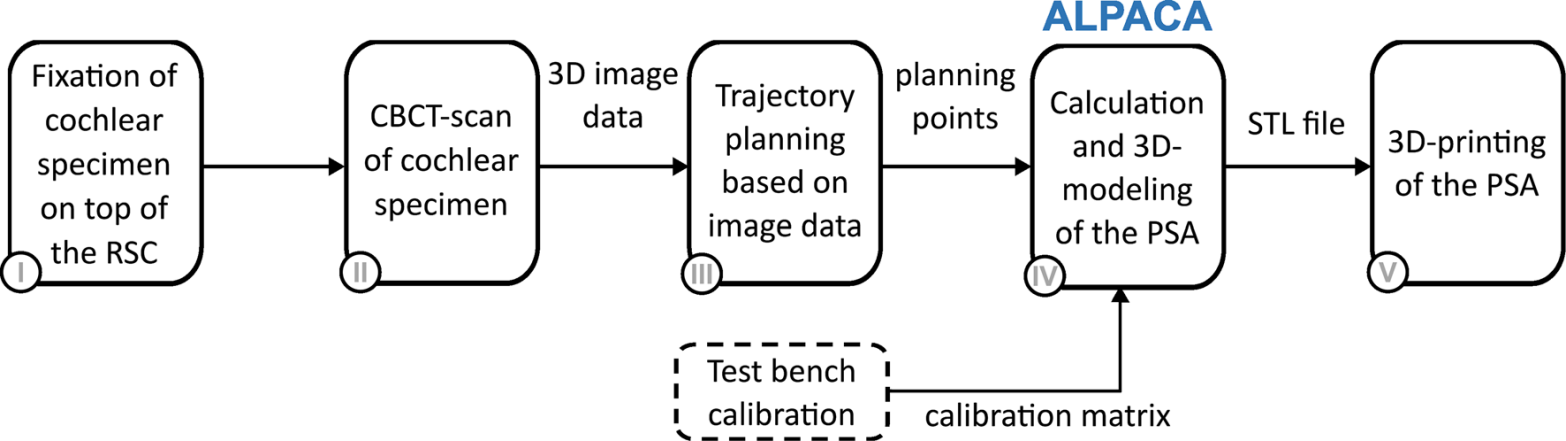
Flowchart of the general workflow for creating an individual PSA

The creation of an individual PSA can be divided into five steps (Fig. 2). First, a dissected cochlear specimen is fixed to a self-designed registration and specimen carrier (RSC). In step two, a cone beam computed tomography (CBCT) scan is performed to acquire volumetric image data of the cochlear specimen and the connected RSC. The image data are imported into a custom planning software in step three, where a registration process is performed using fiducials at the RSC, followed by manual planning of the desired insertion trajectory into the cochlea.

A newly developed algorithm (*ALPACA*—alignment test bench pose setting adapter calculation), implemented in MATLAB (R2022a, The MathWorks, Natick, USA), further processes these planning points (step four). *ALPACA* automatically calculates the PSA geometry and creates a ready-to-print 3D model of it. Additionally, the algorithm can incorporate a calibration matrix to compensate inaccuracies caused, e.g., by manufacturing uncertainties of the test setup. Finally, the output of the program is an STL file of the PSA, which can be sliced and 3D printed with any common slicing program and 3D printer (step five).

### Dissection of porcine cochlear specimen

**Fig. 3:**
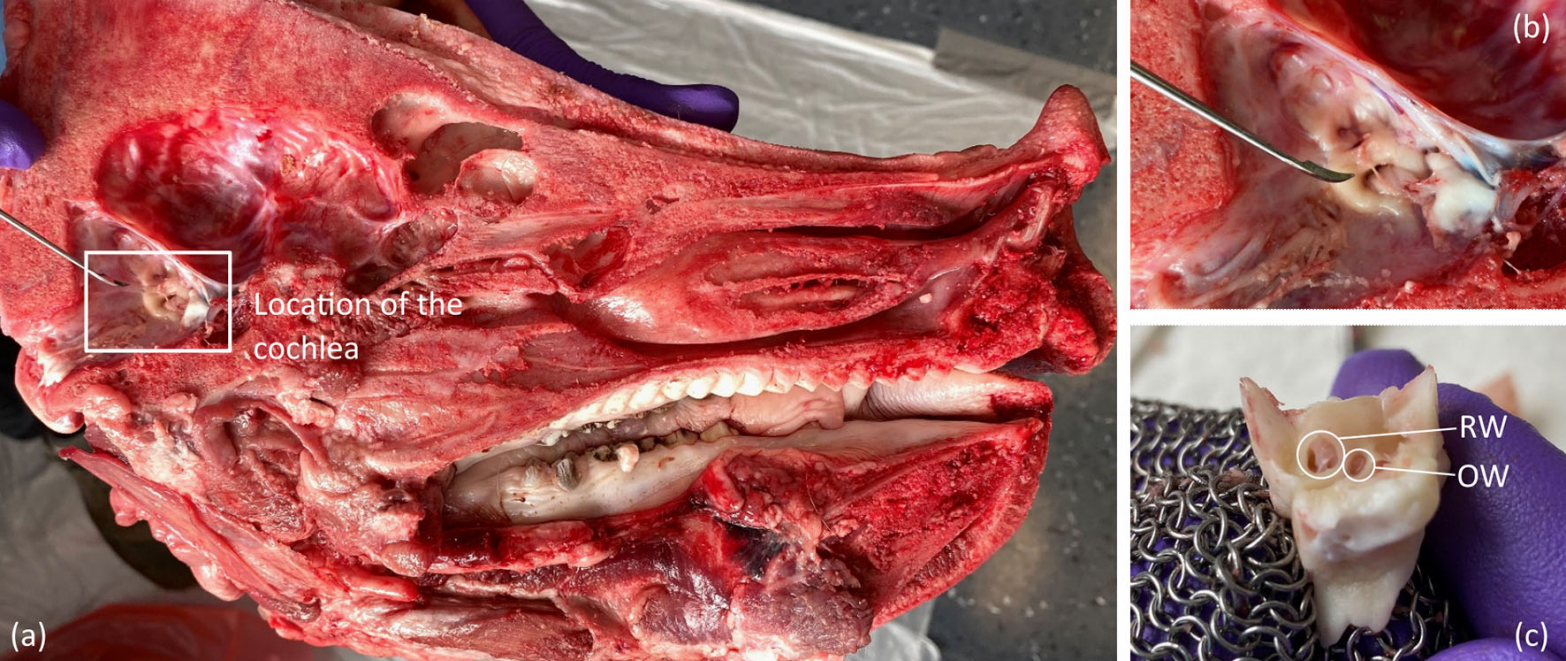
Dissection process: **a** fresh and never-frozen porcine half skull with visible location of the cochlea without further dissection; **b** close-up of the otic capsule within the skull and **c** dissected otic capsule including the cochlea with visible round window (RW) and oval window (OW)

Porcine cochlear specimens, which are part of the otic capsule, were used. These specimens are inexpensive, easy to purchase and similar to human cochleae [22]. The porcine heads were delivered as half skulls (Fig. 3a), where the location of the cochlea can be identified easily without further preparation (Fig. 3b). A band saw was used for large-scale removal of bone and tissue around the identified location of the otic capsule. The remaining tissue was removed using a common surgical forceps and a surgical knife. After sufficient tissue removal, the otic capsule, which measures approx. 15 mm × 15 mm × 15 mm, could carefully be levered out of the surrounding temporal bone. This is possible due to the difference in bone structure between the temporal bone and the otic capsule, which introduces a weak point at the contact surface. An example of a dissected cochlear specimen with visible round and oval window is shown in Fig. 3c.

### Test setup and component design

**Fig. 4:**
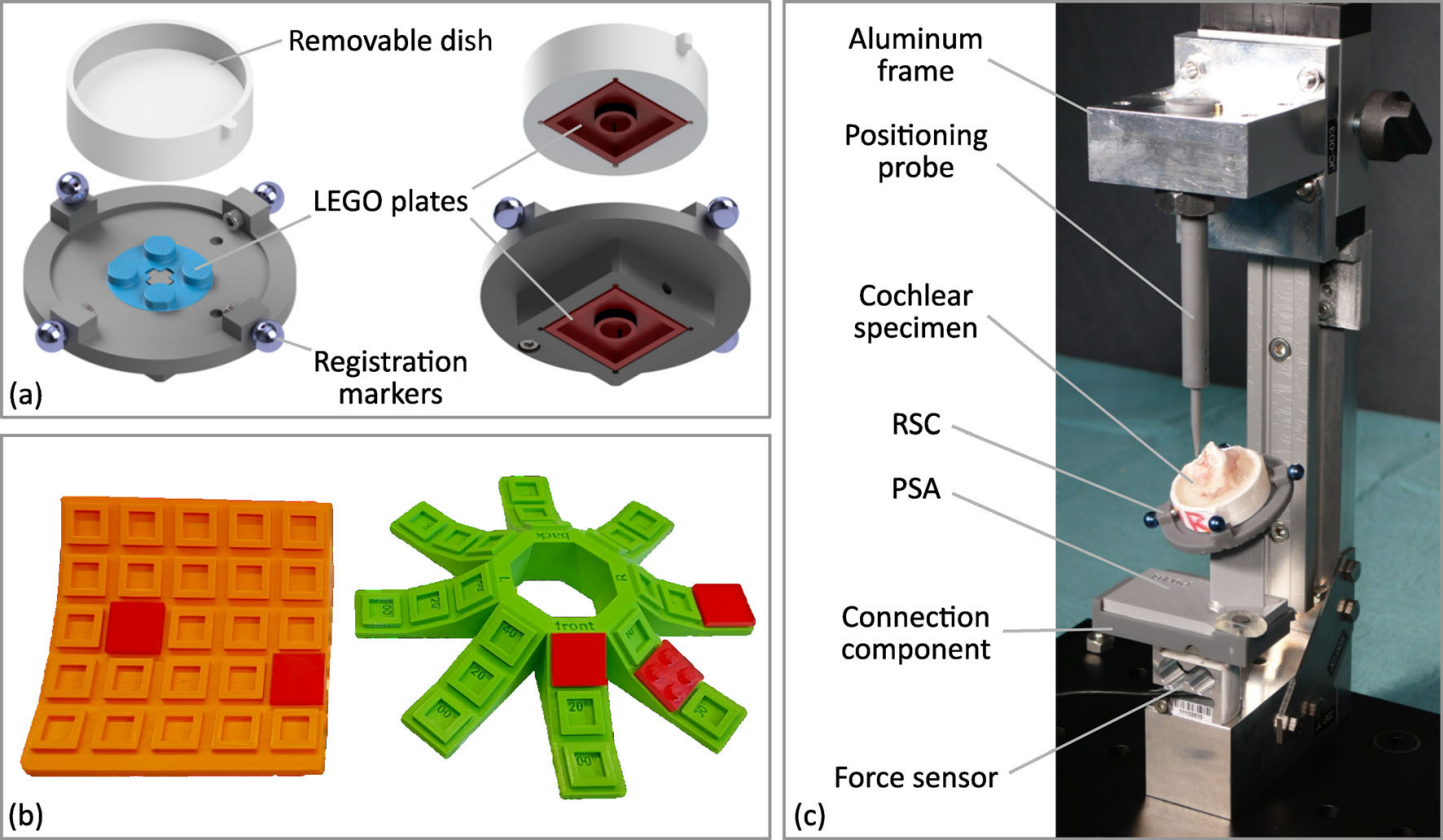
Test setup design: **a** exploded views of the RSC CAD model with its components; **b** evaluation of 3D-printed LEGO connection using varying clearances and angles (left) and by comparing different printing orientations on the build plate of the 3D printer (right) and **c** design of the test setup

The specimens were glued (Paladur®, Heraeus, Hanau, Germany) into a removable dish that acts as an interface as it can be connected to the registration and specimen carrier (RSC). For registration purpose, the RSC includes four titanium spheres with a diameter of 5 mm (Fig. 4a). Furthermore, the RSC provides two interfaces: one to clamp the removable dish on top; the other interface is located at the bottom to connect the RSC to the PSA. Both interfaces are realized using LEGO plates (part no. 3022 and 3068, Lego A/S, Billund, Denmark) to allow the reuse of the RSC and to enable a tight and accurate connection.

In contrast with the RSC, which can be reused, the PSA is individualized for every single specimen. In order to simplify the workflow, the PSA provides a 3D-printed interface, which is optimized to precisely connect it to the LEGO plate at the bottom of the RSC. An evaluation of the connection was performed by printing the interface in slightly different fitting dimensions, i.e., clearance, exact and oversize fit (up to ± 0.1 mm), and varying angles in the expected range (from 0° up to 40°) (Fig. 4b). Additionally, the connections were printed in varying directions to qualitatively investigate, whether the angular position on top of the build plate of the 3D-printer has any effect on the fitting results. The clamping mechanism was examined qualitatively by manually attaching and detaching the LEGO bricks to the printed interface and assessment of the clutch power.

At the bottom side, the PSA can be fixed on top of a connection component to the force sensor (KD24S, ME Messsysteme, Hennigsdorf, Germany) by using a wing nut with a pitch which presses the chamfer on the PSA toward the bottom plate. The whole test setup assembly is shown in Fig. 4c. It consists of a positioning probe, which simulates the insertion axis of the insertion tool for the positioning accuracy evaluation. The probe is connected to the force sensor by a C-shaped aluminum frame, which ensures coaxial alignment of the insertion axis and the force measurement direction.

### Planning of the trajectory into the cochlea

**Fig. 5:**
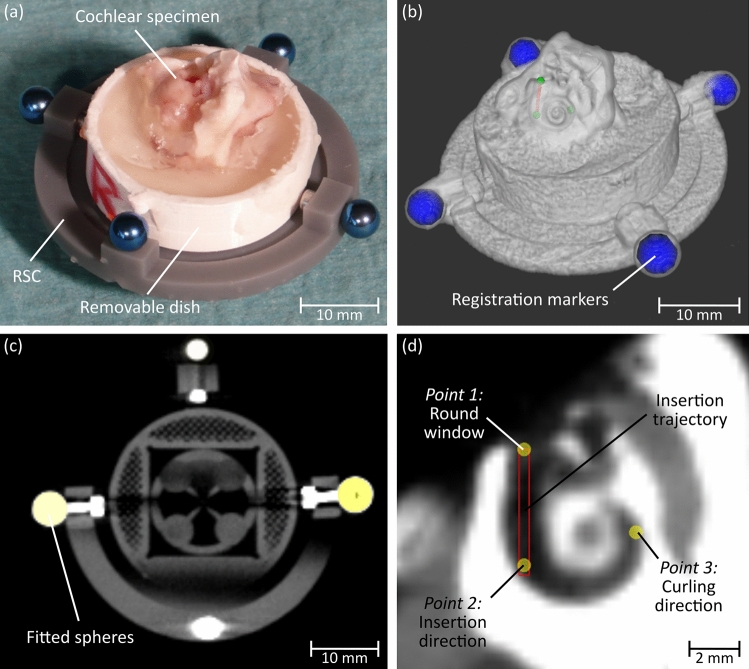
Trajectory planning: **a** Cochlear specimen glued inside a removable dish and clamped on top of the RSC; **b** CBCT data of the specimen on top of the RSC; **c** sphere fitting into the registration markers and **d** planning points for PSA calculation

Processing of the volumetric image data of the specimen on top of the RSC (Fig. 5a) was performed with a custom planning software from our workgroup, which enables semi-automated detection and fitting of the spherical registration markers (Fig. 5b and c) and subsequently performs an automated point-based registration. To avoid a high registration error due to manufacturing inaccuracies of the RSC, the registration process uses the exact positions of the marker centers and the bottom LEGO plate. These were measured in advance using an optical coordinate measurement machine (CMM, XM-1200, KEYENCE, Osaka, Japan) with a measurement accuracy of ± 3 µm.

In total, three planning points are necessary for PSA computation (Fig. 5c):

- *Point 1*: It is located at the center of the round window defining the entrance into the cochlea.
- *Point 2*: It defines the insertion trajectory together with the first point. It is chosen in a way that the resulting trajectory provides a tangential path to the basal turn of the cochlea and stays within the central region of the scala tympani cross section.
- *Point 3*: It defines the curling direction of the cochlea to enable a standardized orientation of the basal turn within the test setup. It is placed approx. at three quarters of the first cochleaer turn and ensures that the rotational orientation of the EA toward the inner axis (modiolus) of the cochlea stays the same throughout every insertion trial.

### Calculation and PSA modeling

**Fig. 6:**
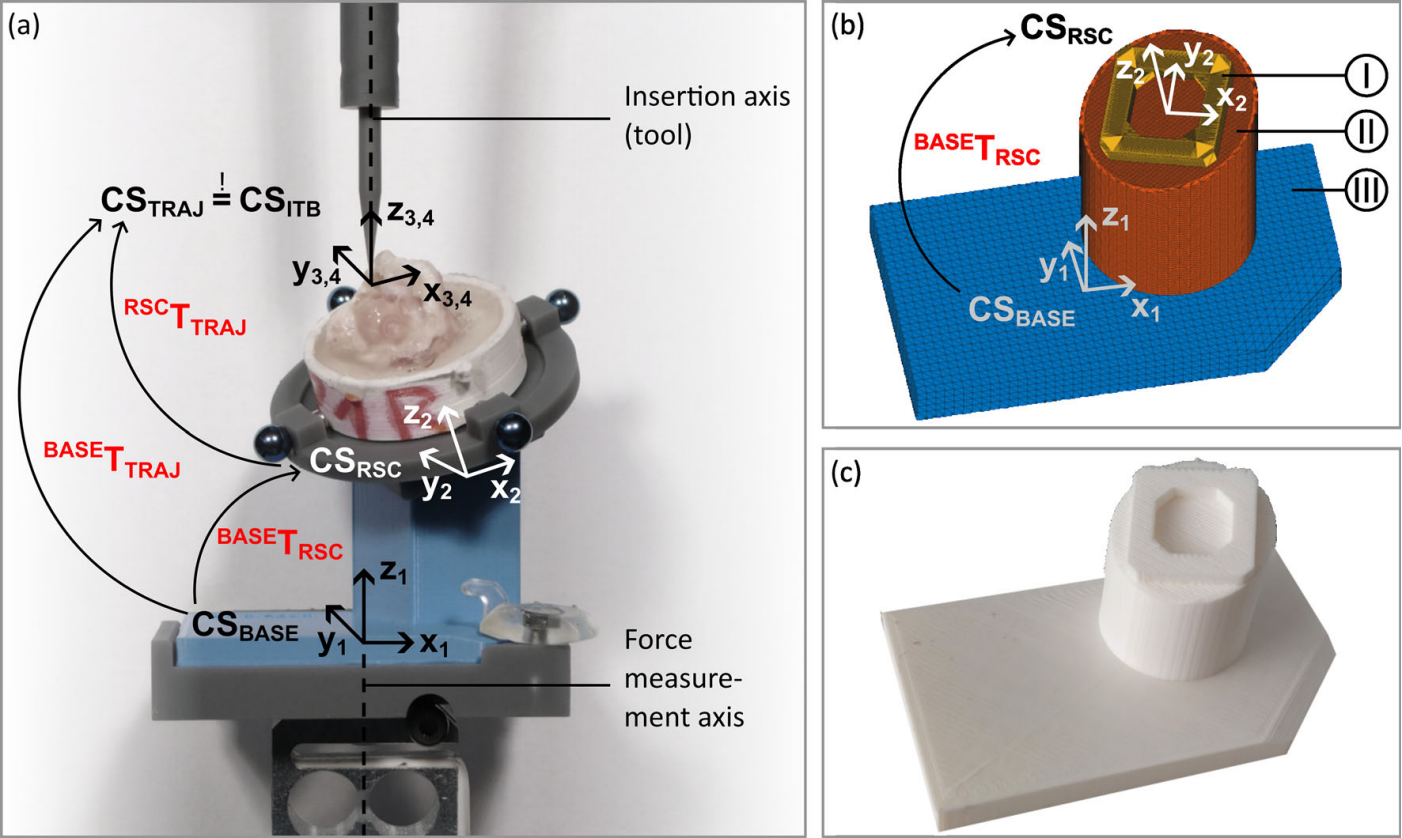
PSA modeling: **a** visualization of the different coordinate systems and the transformation matrices; **b** mathematical alphaShape model of the PSA with its sub-models ‘connector sub-model’ [I], ‘cylinder sub-model’ [II] and ‘base sub-model’ [III] and **c** 3D-printed PSA

The calculations in *ALPACA* are based on homogeneous transformation matrices, which needed definition of four coordinate systems (CS) (Fig. 6a). A more detailed description of the CS is provided in [21]. The goal of the calculation is the mathematical relation between ${\mathrm{CS}}_{\mathrm{BASE}}$ and ${\mathrm{CS}}_{\mathrm{RSC}}$, i.e., the transformation matrix $^{{{\text{BASE}}}} T_{{{\text{RSC}}}}$, since these two CS form the functional surfaces of the pose setting adapter. This matrix is given as

1 $$^{{{\text{BASE}}}} T_{{{\text{RSC}}}} = \,^{{{\text{BASE}}}} T_{{{\text{TRAJ}}}} \cdot^{{{\text{TRAJ}}}} T_{{{\text{RSC}}}} =\,^{{{\text{BASE}}}} T_{{{\text{TRAJ}}}} \cdot \left( {^{{{\text{RSC}}}} T_{{{\text{TRAJ}}}} } \right)^{ - 1} , $$

where $^{{{\text{RSC}}}} T_{{{\text{TRAJ}}}}$ can be calculated from the image registration process described above, as the location of the planning points, and therefore, ${\mathrm{CS}}_{\mathrm{TRAJ}}$ is known within ${\mathrm{CS}}_{\mathrm{RSC}}$. The other transformation matrix $^{{{\text{BASE}}}} T_{{{\text{TRAJ}}}}$ is also specified as the calibration matrix of the test setup, since it provides information of the relation between the main force measurement direction (*z*_*1*_) and the insertion axis of the tool (*z*_4_), which are aligned coaxially by design of the test setup. Besides the calculation of $^{{{\text{BASE}}}} T_{{{\text{RSC}}}}$, *ALPACA* uses the calibration matrix to compensate possible deviations between these axes simply by adjusting the PSA. For this purpose, the test setup, i.e., the relation between ${\mathrm{CS}}_{\mathrm{BASE}}$ and ${\mathrm{CS}}_{\mathrm{TRAJ}}$, was measured in advance using the CMM.

The mathematical 3D model of the PSA is comprised of three sub-models (Fig. 6b):

- *Connector sub-model*: Frame structure to provide a connection to the LEGO plate of the RSC. It is orientated using ${\mathrm{CS}}_{\mathrm{RSC}}$ as center point of the frame and *x*_2_ and *y*_*2*_ as the direction of the frame borders.
- *Base sub-model*: It stays the same in every PSA. It consists of a cuboid base structure and a chamfer for the fixation with a wing nut. ${\mathrm{CS}}_{\mathrm{BASE}}$ is defined at the bottom center of the cuboid.
- *Cylinder sub-model*: It connects the *base sub-model* with the *connector sub-model*. The lower center of the cylinder is located at the projected origin of ${\mathrm{CS}}_{\mathrm{RSC}}$ along *z*_1_ on top of the *base sub-model* cuboid surface.

All sub-models are implemented as point clouds, which are converted to a solid object using the *alphaShape* function provided in MATLAB. After the virtual solid object of the PSA is finalized, *ALPACA* converts the 3D object to a ready-to-print STL file. An example of a 3D-printed PSA is shown in Fig. 6c.

## Experimental evaluation

### Pose setting accuracy

Fifteen porcine cochlear specimens (nine right and six left sides) were dissected as described in order to determine alignment accuracy of the presented method. Image data were acquired using a CBCT device with a voxel size of 300 µm (xCAT, Xoran Technologies LLC, Ann Arbor, Michigan). Three-dimensional printing of the PSA was performed with an FDM printer (Ultimaker 2 + , Ultimaker, Geldermalsen, Netherlands) with a set layer height of 0.1 mm and a nozzle diameter of 0.4 mm.

We evaluated the experiments by measuring the insertion axis of the test setup and the registration markers of the RSC with the CMM after the PSA was mounted between the force sensor and the RSC. With an additional registration step using the measured markers, the relation of the planned trajectory to the actual insertion axis could be determined. Pose accuracy was evaluated by calculating the angular deviation between the planned trajectory and the insertion axis of the test setup and additionally the Euclidean distance between these axes at the level of the round window. For evaluation of the registration process between measured registration marker position and location of the marker within the image data, the fiducial registration error (FRE) was determined.

### Insertion study

To assess the suitability of the presented method for use within an insertion test procedure, the last four cochlear specimens from the pose setting experiments were used for electrode insertion. For this purpose, a linear stage (LTM 45-110-HiSM, OWIS, Staufen im Breisgau, Germany) was added to the test setup to provide a steady forward movement and a measurable insertion depth. A commercial straight EA (SlimStraight, Cochlear Ldt., Sydney, Australia) was partially inserted with 0.1 mm/s up to 17 mm or until the EA started to buckle. We chose 17 mm as the maximum insertion depth due to a preliminary test insertion into another porcine specimen, where the EA could not be inserted further, which was not part of this study. In total, we repeated the EA insertion three times per specimen. After the last insertion into each specimen, an additional CBCT scan was performed to assess the intracochlear position of the EA.

## Results

The creation of the PSAs was successful for all 15 porcine cochlear specimens indicating that the available PSA workspace is sufficient. Three-dimensional printing of the PSA took about 45–60 min depending on the individual geometry. Using the test components (Fig. 4b), the clutch power of the 3D-printed interface to the LEGO brick proved to be quantitively multiple orders of magnitude higher than the expected insertion force when using the exact dimensions of the plate without clearance or overfitting (± 0 mm). An angle dependency of the 3D-printed connection could not be determined in the tested range up to 40°. The tightness of the connection was also independent of the rotational orientation on top of the build plate of the 3D printer.

### Pose setting accuracy

Results of the positioning accuracy are summarized in Table 1. The data show the Euclidean distance between insertion axis and planned trajectory on the level of the round window of ≤ 0.38 mm in all cases, resulting in a mean value of 0.21 mm ± 0.10 mm. For the angular alignment between the axes, the deviation was found to be 0.43° ± 0.21° (max.: 0.9°). For the registration process, a mean fiducial registration error (FRE) of 0.04 mm ± 0.01 mm was found.

**Table 1:** Accuracy values measured in the positioning experiments

Specimen	Side	FRE [mm]	Euclidean distance [mm]	Angular deviation [°]
#01	*R*	0.05	0.10	0.48
#02	*R*	0.05	0.38	0.29
#03	*R*	0.04	0.22	0.50
#04	*L*	0.04	0.19	0.76
#05	*R*	0.04	0.11	0.50
#06	*R*	0.04	0.16	0.21
#07	*R*	0.04	0.10	0.51
#08	*L*	0.04	0.23	0.28
#09	*L*	0.04	0.20	0.08
#10	*R*	0.05	0.05	0.38
#11	*R*	0.04	0.18	0.53
#12	*L*	0.05	0.31	0.30
#13	*L*	0.05	0.28	0.90
#14	*L*	0.05	0.30	0.34
#15	*R*	0.05	0.33	0.42
Mean ± SD		0.04 ± 0.01	0.21 ± 0.10	0.43 ± 0.21

*SD* standard deviation and *FRE* fiducial registration error

### Insertion study

**Fig. 7:**
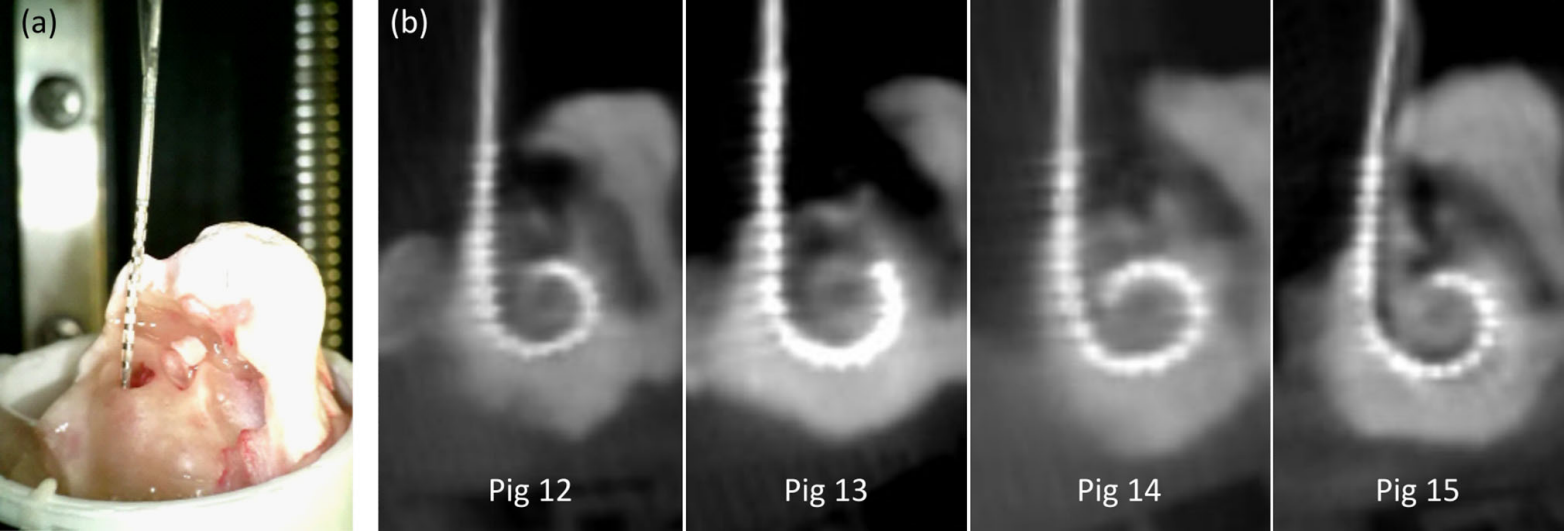
Insertion study: **a** photo of the EA insertion and **b** CBCT scan of the porcine specimens with inserted EA

The insertion into the aligned specimens was possible in all four cases with the EA located correctly above the round window. An insertion up to 17 mm was not possible in any insertion, since the EA started to buckle between 14- and 16-mm insertion depth. Figure 7 shows the beginning of the insertion process as well as the CBCT scans of the inserted EAs. Besides the limited insertion depth, the CBCT data showed typical locations of the EA inside the cochlea, and no abnormalities, e.g., tip foldover or intracochlear buckling, were detected.

## Discussion

### Method and test setup

*ALPACA* proves to be a simple, intuitive and fast way to calculate individual pose setting adapters right after planning the trajectory into the cochlea. Compared to stereo-optic or magnetic navigation systems, our method is rather inexpensive, since only a consumer level 3D printer is needed. Although a CMM was used in our study to determine the calibration matrix, it can be omitted if the experimental setup and the registration carrier are manufactured with sufficient precision. Another advantage of our method is the fixation of the specimen. Using a navigation-based system, the alignment requires intricate manual fine-tuning, and the specimen or the insertion tool needs to be fixed after the alignment, which makes this process complex and time consuming. Due to the modular design with plug-in connections in our test setup, the assembly process is quick and easy, and the fixation is stable. Nevertheless, there is potential for optimizing the interface to the LEGO bricks, as the used bricks allow attachment in four different orientations. In our trials, the knowledge about the correct positioning was obtained from the image data of the previous planning procedure. However, a different brick shape of the connection, e.g., an L-shaped brick, could prevent user errors. The outcome of 0.21 mm ± 0.10 mm Euclidean and 0.43° ± 0.21° angular deviation is similar compared to our previous work (0.23 mm ± 0.12 mm and 0.38° ± 0.17°) [21], with the extension that in this study, we used the *ALPACA* program and positioned real cochlear specimens instead of virtual trajectories for a more realistic application.

### Euclidean distance

To classify the outcome of the Euclidean distance deviation of our method, the results can be compared to benchmark values regarding the target accuracy in computer-assisted cochlear implant surgery. First, Schipper et al. mentioned in 2004 an aimed target region accuracy of ± 0.25 mm for performing a precise cochleostomy using computer-assisted navigation tools [23]. Rau et al. analyzed morphological image datasets of eight human temporal bones in 2019 to determine a target accuracy range for a safe and atraumatic opening of the cochlea by using minimal invasive surgery and came up with a threshold value of ± 0.3 mm [24]. According to these two benchmark values, our results are below the suggested value of Schipper et al. in 10 of 15 cases and compared to the value mentioned by Rau et al. even in 12 of 15 cases, showing a high positioning accuracy of our method. Even our worst-case value of 0.38-mm shift is only slightly out of the discussed range. In addition, our results need to be put into perspective, since we do not perform minimal invasive surgery in our test setup so that the benchmark values should only act as evidence for the sufficiently high accuracy of the presented method.

**Fig. 8:**
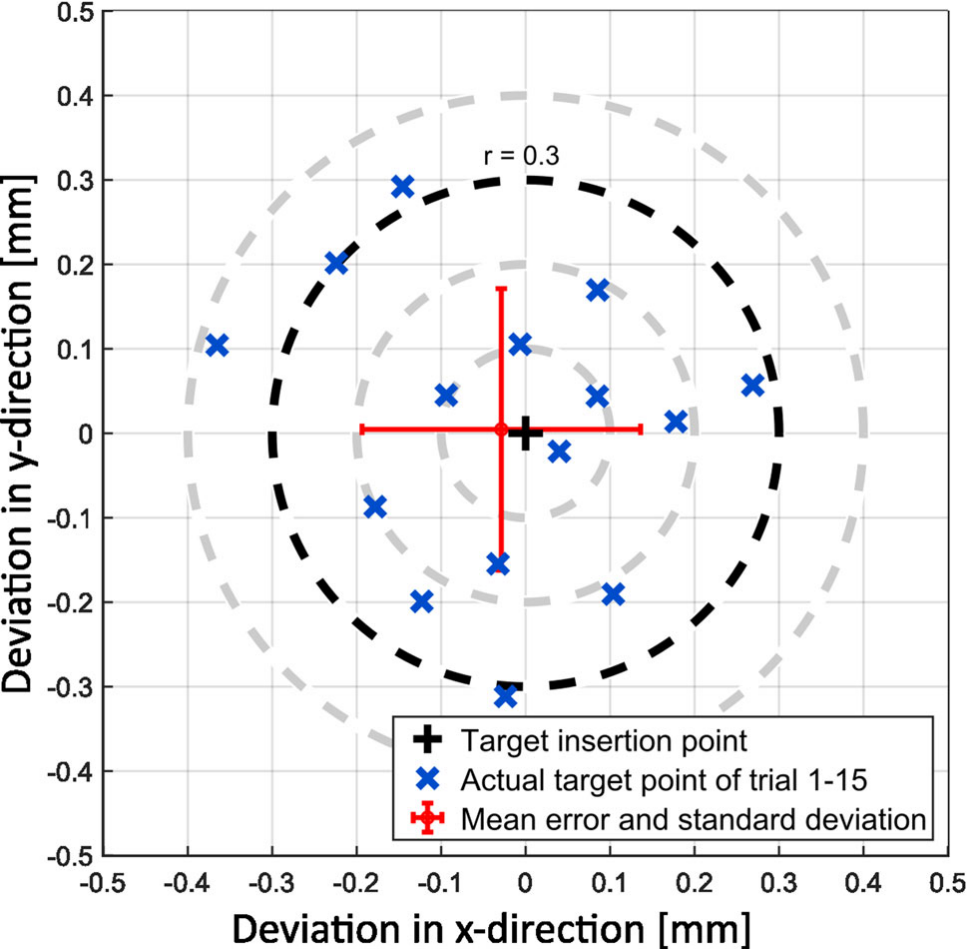
Measured target points at the level of the round window

When assessing the distance between the achieved insertion trajectories and the target trajectories at the level of the round window, the deviations are evenly distributed around the target point (Fig. 8). This behavior can be attributed to the performed test bench calibration measurement, which eliminates systematical errors. Resulting deviations may be caused by manufacturing tolerances of the 3D printing process of the PSA. In our study, we did not investigate other and more accurate 3D printing methods, which might improve accuracy. However, this would also increase the overall costs and from our findings, a consumer level FDM printer is sufficient for the desired application. Another source of error could be the connecting interfaces, as the LEGO bricks might not snap completely into the final position. Redesigning the RSC could allow a better view on the connection in order to provide a better visual control of the remaining gap. The error due to the fiducial registration process only had a minor influence on the alignment accuracy, as indicated by the small FRE (0.04 mm ± 0.01 mm).

### Angular deviation

The spatial orientation of a specimen is more critical than its positioning since the cochlear lumen is not visible from outside. Moreover, a misalignment between the insertion axis and a planned trajectory, ideally placed tangential to the centerline of the basal part of the scala tympani, was shown to increase the insertion forces [4] and to raise the risk of traumatic EA insertions [5]. Both would affect the EA insertion and would lead to a possible misinterpretation of the insertion test results, which makes it essential to minimize this error. Torres et al. showed that aligning the insertion axis toward a planned trajectory into the cochlea by hand is strongly dependent on the experience of the surgeon [17]. In another work of the same group, different alignment procedures were compared, yielding a misalignment between the insertion axis and the planned trajectory of 8.3° ± 2.82°, if the alignment was performed manually by ENT residents, 8.6 ± 2.83° with additional help of an electromagnetic navigation system and 3.4° ± 1.56° when performing the alignment with a robot-based and semi-automated procedure [25]. By using our newly developed method, we achieved a mean angular deviation of 0.43° ± 0.21°, which is 19 times lower than the alignment by hand of a CI expert and even almost 9 times lower than the semi-automated robot-assisted alignment procedure reported by Torres et al. We conclude that our alignment procedure provides a comparably high positioning accuracy and is more suitable for the use of aligning cochlear specimens for insertion tests than manual alignment.

### Insertion tests

The four insertions proved the suitability of the alignment method for the use in insertion tests, as the insertion was possible in all cases, indicating an accurate specimen position. The torque acting on the brick interfaces, induced by the insertion force and the lever arm, did not show a visible influence on the connection. This might be due to the small insertion forces in tenths of a mN range and the comparably tight brick connection. If future studies, e.g., with different specimens, showed that this connection is not solid enough, bricks from other manufacturers could be tested having higher clutch power. The early EA buckling, which was observed between 14- and 16-mm insertion depth, suggests optimization potential in the experimental setup. This limitation might be caused by design of our insertion device, holding the EA too far behind the last electrode contact, which increases the unguided area of the EA and favors buckling. To reduce buckling, a guide tube could be added to the test setup, as shown in other works [4]. An additional limiting factor is the use of only one EA for all experiments, which might have had an effect on the insertion behavior. Another relevant factor is the use of an EA made for human anatomy for the insertion into porcine specimens. Even though the porcine cochlear anatomy is said to be similar to the human one it differs, e.g., in the number of turns (3.5 turns compared to 2.5 turns in humans) [22]. However, when considering the A- and B-values of the porcine cochlear specimens, which are measures of the length and width of the cochlear base [26], it is noticeable that these values are smaller than these of humans. For the porcine specimens, we found a mean *A*-value of 7.31 mm ± 0.15 mm and a mean *B*-value of 5.30 mm ± 0.23 mm (Table 2) compared to human values for *A* between 8.44 and 9.23 mm and for *B* between 6.22 and 7.0 mm, respectively [27]. This could also explain the insertion depth limitation due to a tighter winding of the cochlea and a shorter basal turn.

**Table 2:** *A*- and *B*-values of the porcine cochlear specimens

Pig #	1	2	3	4	5	6	7	8	9	10	11	12	13	14	15	Mean ± SD
*A* [mm]	7.39	7.38	7.09	7.45	7.30	7.28	7.22	7.31	7.30	6.98	7.21	7.32	7.57	7.30	7.54	7.31 ± 0.15
*B* [mm]	5.47	5.34	5.01	5.24	5.3	5.54	5.57	5.39	5.2	4.71	5.17	5.37	5.37	5.14	5.70	5.30 ± 0.23

*SD* standard deviation

Nevertheless, porcine cochlear specimens are a promising alternative to human temporal bones for future insertion tests, since the rather short insertion depth might be sufficient for several research questions. For instance, investigation of intracochlear frictional conditions and the influence of EA coatings or different insertion trajectories on the insertion forces are current research questions [4, 11, 28–30]. Here, even partial insertions could improve the understanding of mechanical interrelationships. Even more: Contrary to most human cadavers used for experiments, porcine specimens are available in a very fresh and never-frozen condition, which might be as close to intraoperative reality as possible in terms of mechanical properties, such as intracochlear friction. Therefore, the use of porcine specimens for EA insertion tests should be further investigated in future studies. Nevertheless, if realistic anatomical dimensions were required, the presented alignment method could also be used for human cochlear specimens with only small necessary adjustments, such as the enlargement of the removable dish.

## Conclusion

In summary, we presented a new advanced methodology for the alignment of cochlear specimens with respect to a desired insertion trajectory using 3D-printed pose setting adapters (PSA). For this purpose, an algorithm enabling the automated generation of a ready-to-print 3D model of individual PSAs from planning data was implemented in MATLAB. Alignment and insertion experiments using porcine cochlear specimens demonstrated a high precision of the method and proved the feasibility of the whole workflow. The presented method decreases error sources in EA insertion tests using cochlear specimens, allowing for a higher level of standardization. This increases the repeatability of insertion experiments and thereby the comparability of experimental results.

## Electronic supplementary material

Below is the link to the electronic supplementary material.

Supplementary file1 (M 40 kb)

Supplementary file2 (M 9 kb)

Supplementary file3 (STP 374 kb)

Supplementary file4 (TXT 35 kb)

Supplementary file5 (STP 1273 kb)

## Data Availability

The *ALPACA* algorithm and associated STEP files are available in the supplemental material of this work, provided by the authors.

## References

[1] Gantz BJ, Turner C, Gfeller KE, Lowder MW (2005) Preservation of hearing in cochlear implant surgery: advantages of combined electrical and acoustical speech processing. Laryngoscope 115:796–802. 10.1097/01.MLG.0000157695.07536.D2 15867642 10.1097/01.MLG.0000157695.07536.D2

[2] Lin C-C, Chiu T, Chiou H-P, Chang C-M, Hsu C-J, Wu H-P (2021) Residual hearing preservation for cochlear implantation surgery. Tzu Chi Med J 33:359. 10.4103/tcmj.tcmj_181_20 34760631 10.4103/tcmj.tcmj_181_20 PMC8532579

[3] Bas E, Dinh CT, Garnham C, Polak M, Van de Water TR (2012) Conservation of hearing and protection of hair cells in cochlear implant patients’ with residual hearing. Anat Rec 295:1909–1927. 10.1002/ar.22574 10.1002/ar.22574 23044907

[4] Aebischer P, Mantokoudis G, Weder S, Anschuetz L, Caversaccio M, Wimmer W (2022) In-vitro study of speed and alignment angle in cochlear implant electrode array insertions. IEEE Trans Biomed Eng 69:129–137. 10.1109/TBME.2021.3088232 34110987 10.1109/TBME.2021.3088232

[5] Torres R, Drouillard M, De Seta D, Bensimon JL, Ferrary E, Sterkers O, Bernardeschi D, Nguyen Y (2018) Cochlear implant insertion axis into the basal turn: a critical factor in electrode array translocation. Otol Neurotol 39:168–176. 10.1097/MAO.0000000000001648 29194215 10.1097/MAO.0000000000001648

[6] De Seta D, Torres R, Russo FY, Ferrary E, Kazmitcheff G, Heymann D, Amiaud J, Sterkers O, Bernardeschi D, Nguyen Y (2017) Damage to inner ear structure during cochlear implantation: correlation between insertion force and radio-histological findings in temporal bone specimens. Hear Res 344:90–97. 10.1016/j.heares.2016.11.002 27825860 10.1016/j.heares.2016.11.002

[7] Mirsalehi M, Rau TS, Harbach L, Hügl S, Mohebbi S, Lenarz T, Majdani O (2017) Insertion forces and intracochlear trauma in temporal bone specimens implanted with a straight atraumatic electrode array. Eur Arch Oto-Rhino-Laryngol 274:2131–2140. 10.1007/s00405-017-4485-z 10.1007/s00405-017-4485-z 28238160

[8] Kaufmann CR, Henslee AM, Claussen A, Hansen MR (2020) Evaluation of insertion forces and cochlea trauma following robotics-assisted cochlear implant electrode array insertion. Otol Neurotol 41:631–638. 10.1097/MAO.0000000000002608 32604327 10.1097/MAO.0000000000002608

[9] Nguyen Y, Miroir M, Kazmitcheff G, Sutter J, Bensidhoum M, Ferrary E, Sterkers O, Bozorg Grayeli A (2012) Cochlear implant insertion forces in microdissected human cochlea to evaluate a prototype array. Audiol Neurotol 17:290–298. 10.1159/000338406 10.1159/000338406 22653365

[10] Kobler J-P, Beckmann D, Rau TS, Majdani O, Ortmaier T (2014) An automated insertion tool for cochlear implants with integrated force sensing capability. Int J Comput Assist Radiol Surg 9:481–494. 10.1007/s11548-013-0936-1 23959671 10.1007/s11548-013-0936-1

[11] Aebischer P, Caversaccio M, Wimmer W (2021) Fabrication of human anatomy-based scala tympani models with a hydrophilic coating for cochlear implant insertion experiments. Hear Res 404:108205. 10.1016/j.heares.2021.108205 33618163 10.1016/j.heares.2021.108205

[12] Hügl S, Rülander K, Lenarz T, Majdani O, Rau TS (2018) Investigation of ultra-low insertion speeds in an inelastic artificial cochlear model using custom-made cochlear implant electrodes. Eur Arch Oto-Rhino-Laryngol 275:2947–2956. 10.1007/s00405-018-5159-1 10.1007/s00405-018-5159-1 30302574

[13] Rau TS, Hussong A, Leinung M, Lenarz T, Majdani O (2010) Automated insertion of preformed cochlear implant electrodes: evaluation of curling behaviour and insertion forces on an artificial cochlear model. Int J Comput Assist Radiol Surg 5:173–181. 10.1007/s11548-009-0299-9 20033522 10.1007/s11548-009-0299-9

[14] Zuniga MG, Hügl S, Engst BG, Lenarz T, Rau TS (2021) The effect of ultra-slow velocities on insertion forces: a study using a highly flexible straight electrode array. Otol Neurotol 42:E1013–E1021. 10.1097/MAO.0000000000003148 33883518 10.1097/MAO.0000000000003148

[15] Van Der Marel KS, Briaire JJ, Wolterbeek R, Snel-Bongers J, Verbist BM, Frijns JHM (2014) Diversity in cochlear morphology and its influence on cochlear implant electrode position. Ear Hear 35:9–20. 10.1097/01.aud.0000436256.06395.63 10.1097/01.aud.0000436256.06395.63 24196418

[16] Meng J, Li S, Zhang F, Li Q, Qin Z (2016) Cochlear size and shape variability and implications in cochlear implantation surgery. Otol Neurotol 37:1307–1313. 10.1097/MAO.0000000000001189 27579839 10.1097/MAO.0000000000001189

[17] Torres R, Kazmitcheff G, Bernardeschi D, De Seta D, Bensimon JL, Ferrary E, Sterkers O, Nguyen Y (2016) Variability of the mental representation of the cochlear anatomy during cochlear implantation. Eur Arch Oto-Rhino-Laryngol 273:2009–2018. 10.1007/s00405-015-3763-x 10.1007/s00405-015-3763-x 26324880

[18] Yasin R, Dedmon M, Dillon N, Simaan N (2019) Investigating variability in cochlear implant electrode array alignment and the potential of visualization guidance. Int J Med Robot Comput Assist Surg. 10.1002/rcs.2009 10.1002/rcs.2009 31099146

[19] Smetak MR, Riojas KE, Sharma RK, Labadie RF (2022) Beyond the phantom: unroofing the scala vestibuli in a fresh temporal bone as a model for cochlear implant insertion experiments. J Neurosci Methods 382:109710. 10.1016/j.jneumeth.2022.109710 36207005 10.1016/j.jneumeth.2022.109710

[20] Hügl S, Henke M, Kahrs L, Ortmaier T, Lenarz T, Rau T (2019) Development of a test bench for insertion force measurements with precise orientation of specimen using a stereo optical navigation system. Laryngorhinootologie 98(S02):316. 10.1055/s-0039-1686402

[21] Rau TS, Cramer J, Zuniga MG, Böttcher G, Lenarz T (2021) A method for image-guided positioning of cochlear specimens in insertion test benches using 3D printed stands. Curr Dir Biomed Eng 7:105–108. 10.1515/cdbme-2021-2027

[22] Knoll RM, Reinshagen KL, Barber SR, Ghanad I, Swanson R, Smith DH, Abdullah KG, Jung DH, Remenschneider AK, Kozin ED (2019) High resolution computed tomography atlas of the porcine temporal bone and skull base: anatomical correlates for traumatic brain injury research. J Neurotrauma 36:1029–1039. 10.1089/neu.2018.5808 29969939 10.1089/neu.2018.5808 PMC8349728

[23] Schipper J, Aschendorff A, Arapakis I, Klenzner T, Teszler CB, Ridder GJ, Laszig R (2004) Navigation as a quality management tool in cochlear implant surgery. J Laryngol Otol 118:764–770. 10.1258/0022215042450643 15550181 10.1258/0022215042450643

[24] Rau TS, Kreul D, Lexow J, Hügl S, Zuniga MG, Lenarz T, Majdani O (2019) Characterizing the size of the target region for atraumatic opening of the cochlea through the facial recess. Comput Med Imaging Graph. 10.1016/j.compmedimag.2019.101655 31539862 10.1016/j.compmedimag.2019.101655

[25] Torres R, Kazmitcheff G, De Seta D, Ferrary E, Sterkers O, Nguyen Y (2017) Improvement of the insertion axis for cochlear implantation with a robot-based system. Eur Arch Oto-Rhino-Laryngol 274:715–721. 10.1007/s00405-016-4329-2 10.1007/s00405-016-4329-2 27704279

[26] Escudé B, James C, Deguine O, Cochard N, Eter E, Fraysse B (2006) The size of the cochlea and predictions of insertion depth angles for cochlear implant electrodes. Audiol Neurotol 11:27–33. 10.1159/000095611 10.1159/000095611 17063008

[27] Khurayzi T, Almuhawas F, Sanosi A (2020) Direct measurement of cochlear parameters for automatic calculation of the cochlear duct length. Ann Saudi Med 40:212–218. 10.5144/0256-4947.2020.218 32493102 10.5144/0256-4947.2020.218 PMC7270618

[28] Radeloff A, Unkelbach MH, Mack MG, Settevendemie C, Helbig S, Mueller J, Hagen R, Mlynski R (2009) A coated electrode carrier for cochlear implantation reduces insertion forces. Laryngoscope 119:959–963. 10.1002/lary.20206 19358253 10.1002/lary.20206

[29] Ertas YN, Ozpolat D, Karasu SN, Ashammakhi N (2022) Recent advances in cochlear implant electrode array design parameters. Micromachines 13:1081. 10.3390/mi13071081 35888898 10.3390/mi13071081 PMC9323156

[30] Dohr D, Fiedler N, Schmidt W, Grabow N, Mlynski R, Schraven SP (2021) Frictional behavior of cochlear electrode array is dictated by insertion speed and impacts insertion force. Appl Sci 11:5162. 10.3390/app11115162

